# A Public Health Fund

**Published:** 1920-10-09

**Authors:** 


### A Public Health Fund.

The scheme outlined by Sir Arthur Stanley of the uses to be made by the funds received as the result of the special appeal (October 9 to 14) by Red Cross and allied societies seems wise, and his explanation of the situation timely. To take the second point first, it is a natural inquiry to make of the Red Cross as to what it is doing with the war funds, and Sir Arthur—justly we think—points out that all this money was collected for the needs of the sick and wounded, and must be used, as far as possible, in that connection. A large part of the money subscribed on "Our Day," 1918, has been returned to the counties and dominions subscribing it on condition that it is to be used primarily for the relief of sick and wounded soldiers and sailors, and a certain sum is being retained for possible calls of such men in the next few years. If those calls are not very heavy, then some of the money may be devoted to general peace work. It is difficult to quarrel with a decision of this kind.

So far as the proceeds of this month's appeal are concerned, they will be devoted entirely to home work; 75 per cent. of the money collected in any county will be returned to that county, and the remaining 25 per cent. remitted to headquarters. The county organisations have full discretion as to the expenditure of this money on their various public health schemes. Some counties concentrate on maternity and child-welfare, some on the perfecting of their voluntary hospital system, and others on district home nursing—each of great importance. Among other things, the aim of the Red Cross is that there shall be a national service of district nurses, and it is also going to concentrate in the counties on the provision of convalescent homes, with the conviction that by using these for patients in the early stages of disease health may be restored and the strain on the hospitals relieved. There is no limit to what can be done, except the limit of funds. With the 25 per cent. going to headquarters, Sir Arthur said they had two great objects in view—one is to help the poorer counties, and the other to carry on intensive educational propaganda in regard to public health service.

